# Account of Yellow Fever in New Orleans

**Published:** 1835

**Authors:** E. H. Barton


ART. II.—*Account of the Epidemic Yellow Fever which prevailed in New Orleans, during the autumn of* 1833. By E. H. BARTON, M. D.—The circulation of our journal is in the West, and all the West is connected with NEW ORLEANS. Every thing which relates to the climate and diseases of this modern Alexandria, is of interest to our readers, and we thank Dr. Barton for having sent us a copy of the pamphlet, which, for want of space in the department of Reviews, we are obliged to introduce under our Analytical head.

"New Orleans is situated in latitude 29° 57′, and longitude west from Washington 13° 9′, on the left bank, and in a large bend of the Mississippi. It is ten feet and a half above the level of the sea, from which it is distant in the course of the river about one hundred miles, about sixty miles in a south west, and about forty in an eastward course. Five miles north of it is Lake Ponchartrain, which receives the drainage of the city and superfluous waters of the vicinity through the Bayou, St. John, &c. The inclination from Levee street in front, to Rue Marais in the rear, is about eight feet three inches. The city is fanned by the delightful sea breeze from the south west every morning, which is the harbinger of health whilst the prevalent wind. The river rises about fourteen feet, and generally reaches its maximum elevation late in March or early in April. The level of the city is several feet below high water mark, and it is protected from inundation by an embankment or levée three feet and a half high, which extends about five miles along the river, and about two thirds of a mile back to the swamp. The city is built upon a soft alluvial soil, but a few feet above the water in the wells, (dependent upon the state of the river,) the dampness is consequently very great; the streets are filthy, and but partially and badly paved. In the body of the city, (1-24th of the whole area,) water is made to run constantly along the gutters from the river to the swamps during the summer. In 1830 the city contained about 46,310 inhabitants, exclusive of a floating population during the winter and spring months of from 15 to 20,000; increasing on an average of near 2½ per cent. per annum, and hence containing in 1833, 53,234, making altogether about 70,000 inhabitants." p. 3.

Dr. Barton has appended to his Account an elaborate meteorological register for each month of the year 1833, from May to December inclusive; but as we are not furnished with similar tables for other years, the present can only be of value hereafter, when by his own, or the industry of other observers, corresponding observations shall be made. Thus we are at a loss to know, in what respect the summer and autumn of 1833 differed from those of other years, and, of course, unable to decide how far the epidemic he has described depended on the sensible qualities of the atmosphere.

In January, February, March and April preceding the yellow fever, the prevalent diseases were decidedly intestinal, such as diarrhœa and dysentery, with many cases of epidemic cholera. In May the tendency to the last was still more apparent. By the beginning of June this pestilence was prevalent and reached its height on the 8th.

"When there occurred a heavy fall of rain, with much thunder and lightning, soon after which the wind veered round to the S. W. and W. the disease gradually declined. The epidemic strongly impressed its character upon most diseases of the month, uniting its symptoms with those of other diseases, as cramps, or rice water discharges, with ordinary symptoms of bilious fever—or the peculiar coldness, or the vomiting, with a *great* and *indeed* excessive sensibility of the *alimentary canal*, with liability to run into diarrhœa upon the least change of temperature or transgression in living. This disease was indiscriminate in its attacks, with regard to age, sex, or color, selecting those whose equilibrium of constitution was deranged or thrown from its balance from intemperance or imprudence of *any kind*, or from change of diet—*always* however with premonitory diarrhœa, ranging in its duration from six to forty-eight hours, and running into collapse in from three to twenty hours. The disease finally lost its intensity and prevalence with the changes in the condition of the atmosphere." p. 4.

In July, the city became "pretty healthy," with a great tendency, however, to bowel complaints, and the occurrence of bilious fever, under exposure to changes of weather.

The month of August was not in its average heat quite so warm as July, but the quantity of rain, 8.17 inches, was nearly three times as great, and the thermometer rose to 81, 2, 3 and 4 degs. at midnight, for a part of the month, which was unprecedented.

"Before the middle of the month the wind got round to the eastward, and continued blowing fresh with a high thermometric range, the weather very oppressive and showery, and from the streets there arose a very offensive odour. In the second week the yellow fever broke out, and continued increasing until it reached a daily average of thirty cases. Its type was most malignant, with great determination to the head." p. 5.

In the month of September the disease gradually became more mild and manageable, and towards the end, intermittent fevers, diarrhœa and dysentery made their appearance, and blended their symptoms with those of the declining epidemic.

Through the month of October and November it continued slowly to abate, and in December bronchial irritation and inflammation, were the prevalent diseases with a few cases of scarlatina and cholera.

"As precursory to the dreadful scourge which followed, it becomes me to mention several enlargements of the inguinal glands, occurring without the least suspicion of venereal contamination, similar to those mentioned by authors as premonitory of the existence of mortal epidemics; also several cases of menorrhagia. The streets throughout the city were filthy, exhaled a peculiarly offensive odour after rains, and generally so at night. There was much sickness with horses, cattle, and swine in the country.

"The country throughout was unusually sickly; crops fine; fruit bad. Weather during autumn beautiful, air cool, delightful atmosphere, exhibiting the brightness of an Italian sky, as has been so often remarked during our severe epidemics; early and severe frosts, which destroyed near one-third of the brilliant prospects of the husbandman, combined, with other causes, to make the year the most disastrous, we have ever experienced.

"From the preceding succinct and general medical and meteorological history of the year, it cannot have escaped attention that the precursors of the malignant epidemic of the season were gastro-intestinal affections of every grade and severity, from simple extra-sensibility of the gastro-intestinal mucous membrane, to the malignant cholera running its course in six hours. The evils resulting from this epidemic constitution of the atmosphere, and the predisposition to the malignant yellow fever that afterwards occurred, was greatly aggravated by the adoption of the advice so generally given, to live high and drink freely of brandy, &c. to prevent an attack of cholera." pp. 6—7.

Our author divides the cases of yellow fever which fell under his observation, into three classes.

"Class I. *The Congestive.*—The attack is sudden, the patient is at once prostrated and overwhelmed; there is dull pain in the head; questions are answered with difficulty; skin bronzed; eyes red and muddy, like one drunk; pulse weak, soft, or natural; tongue with red edges or natural; extremities cold, and skin generally so, except the central portions of the body, which are preternaturally hot; pain and oppression in epigastrium; appearance of great anguish, or insensibility; the patient lying uncomplaining on one side or back as if but little ailed him; stupor; pupils enlarged; hæmorrhages. Reaction slow, and requiring much, but cautious, depletion to open the condition.

"Class II. *The open inflammatory form.*—This was the most usual, it commenced generally with a chilliness, followed by violent pain in the lower part of the forehead, just below the eyebrows, and in the back and loins; high fever; pulse from 100 and 125; tongue white with red edges; eyes inflamed and blood-shot, with the peculiar muddy, idiotic expression, with great sensibility of the stomach, and ardent desire for cool drinks; tenderness and tension in epigastrium, running through regular stages.

"Class III. *Simple form.*—This class differs but little from the preceding, except in the general mildness of the symptoms; the pain in the head is more diffused and general; not much or severe pain in back or extremities; the tongue sometimes loaded, edges red and fleshy, and still great sensibility of the alimentary canal to medicines; the type easily recognised by the peculiar expression of the eyes, not so red and injected, but dull and muddy, occurring mostly in the latter part of the season, or in those accustomed to a southern latitude, in whom the predisposition was not strong, and acclimating mild."—pp. 9–10.

We have not space to follow our author through his analysis of the symptoms, and shall limit ourselves to the annunciation of his conclusions on the pathology and therapeutics of the disease.

"From the foregoing observations and physiological explanation of the symptoms, we presume the following corollaries will be admitted, viz:—

"1st. That there are *general symptoms* of *inflammation*.

"2d. That there are symptoms of specific or *local inflammation*.

"3d. That the *primary seat* of this inflammation is in the *stomach*.

"4th. That other organs are but *secondarily* affected, as the brain, liver, &c. Upon this pathology we lay the foundation of our treatment.

"Let us proceed then to the exposition of the principles simplified by this view of the subject, and reduced in it its indication, to—

"1st. Controlling or subduing the general inflammatory disposition of the system, produced by the primary influence of the disease on the most susceptible organ, and one having the greatest range of sympathies.

"2d. Subduing that inflammation itself; and—

"3d. Removing its sympathizing consequences in the organic system, and system of relation.

"The 1st is accomplished by general bleeding.
"The 2d by local bleeding, by cooling drinks, &c.
"The 3d by local detractions of blood, by cold ablutions, ice mucilages, aperient medicines, enemata, pediluvia, and fomentations, &c." —pp. 13–14.

We deem it unnecessary to follow our author through his defence of the treatment here proposed. His reasonings are those of the physiological school, of which, as we have made known on a former occasion, he is one of the most able and zealous disciples, who have as yet put forth the results of their experience in the Valley of the Mississippi. Nor shall we analyze the nine cases, which he has detailed, but proceed at once to the results of his practice:

"Recovered, 69. Died, 6; of these, 1 died from imprudent and unauthorized use of the glyster-pipe by the nurse, after every appearance of recovery. 1 died from hæmorrhage of the gums, in consequence of having taken a large dose of calomel before I was called. 1 died from want of proper attendance—no nurse; room over a nine-pin alley, the noise of which prevented his sleeping at a critical time. 1 from relapse, brought on by strong soup taken on the eighth day."—p. 36.

Well! this must, we think, be acknowledged, by all, to have been successful practice. Six fatal out of seventy-five cases—only one death to twelve and a half recoveries, in a malignant yellow fever—is honorable success—as great success as ought ever to be expected under any plan of treatment. As the method pursued by Dr. Barton, does not seem to have been followed by all the medical gentlemen of New Orleans, we should be much pleased to see an account of the success of the mercurial and purging plans, that we might be fully satisfied as to their comparative efficacy. That of our author is manifestly the least offensive to the patient, and if attended with equal benefits ought to be preferred. We are advocates for the guidance of principle; but medicine is an *a posteriori*, not an *a priori* science, and the correctness of its principles, can only be determined by experiment.

A zealous reformer, Dr. Barton is not content with stating the results of his own method of treatment, but through 12 or

15 of his last pages considers with argument and sarcasm the value of other modes of practice, classing them under three heads—1st, the Mercurial; 2d, the Purgative with moderate bleeding; 3d, the West India or French, by ptisans, diaphoretics, &c. We have read these pages with some attention, but cannot say that we entirely concur with him, in the views which he presents. In reasoning against calomel and cathartics, his data are drawn much more from their abuse than their judicious employment—from their being *substituted* for general and local blood-letting, instead of being used *after* a due resort to those antiphlogistics. When thus preceded, we regard them as not only safe but beneficial. His prohibition proceeds on the assumption, that if there be gastro-enteric inflammation, they *necessarily* do harm in every stage; but we consider them as injurious, only in the early and acute periods of the disease. After the bloodvessels have been emptied to a proper degree, we presume them not merely innocent, but well calculated to finish the cure, by the removal of irritating matter from the *primæ viæ*, repleting the portal circle, depurating the blood, diverting from the head, and superseding the dangerous morbid action, with their own specific and temporary excitement; none of which salutary effects can, however, be obtained while the bloodvessels are plethoric, and the powers of the heart inordinate.

Notwithstanding this difference of opinion with our respectable author, we confess ourselves much pleased with his monograph of the epidemic of 1833. It evinces great industry, an industry which, it has been too often affirmed, is not practicable in hot climates, and the materials are put together with a skill and taste, which presage future labours of much merit. We learn by the title page, that the author is professor of Materia Medica, Therapeutics and Hygeine, in the Medical College of Louisiana; an institution of which we have as yet scarcely heard any thing, and concerning which we should be happy, to be enabled to say something in a future number of this Journal.

